# Primary pulmonary Ewing’s sarcoma: rare cause of massive hemothorax in a young girl-case report

**DOI:** 10.1186/s12887-021-02672-6

**Published:** 2021-04-22

**Authors:** Xuefeng Ling, Jianlin Tong, Liangliang Wang, Chuan Yao, Zhiying Chen

**Affiliations:** 1 Respiratory Medicine Department, Affiliated Hospital of Jiujiang University, Jiujiang, Jiangxi China; 2 Pathology Department, Affiliated Hospital of Jiujiang University, Jiujiang, Jiangxi China; 3 Cardio-thoracic Surgical Department, Affiliated Hospital of Jiujiang University, Jiujiang, Jiangxi China; 4 Jiujiang Clinical Precision Medicine Research Center, Jiujiang, Jiangxi China

**Keywords:** Ewing’s sarcoma, Pulmonary, Hemothorax

## Abstract

**Background:**

Ewing’s sarcoma is a common malignant bone tumor in children and young adults. Rarely, extra-skeletal soft tissues and visceral organs can also be the site of origin of Ewing’s sarcoma. Primary pulmonary Ewing’s sarcoma is an extremely rare malignancy.

**Case presentation:**

We report an unusual case of primary pulmonary Ewing’s sarcoma in a 15-year-old girl who initially presented with massive hemothorax. By histopathology evaluation of surgical biopsy specimens, the diagnosis of extraosseous Ewing’s sarcoma was confirmed by both light microscopy and immunohistochemistry. Emergency, open surgery was performed by thoracic surgery at an early stage. After 3 cycles of chemotherapy, the patient was found to be stable at follow-up examination. No more hydrothorax or other symptoms.

**Conclusions:**

We have described an extremely rare case of primary pulmonary Ewing’s sarcoma with massive hemothorax. The patient underwent surgical resection and postoperative chemotherapy, no sign of recurrence to date as an outcome.

## Background

Ewing’s sarcomas are relatively rare neuroectodermal tumors that primarily arise from the bone. Extraosseous Ewing’s sarcomas are a subset of primitive neuroectodermal tumors, which involve the soft tissues and organs such as the ovary, paravertebral soft tissue, testis, uterus, pancreas, renicapsule, and myocardium [1]. We have analyzed data from relevant studies published in PubMed Central and Excerpta Medica Database, and a total of 25 cases of primary pulmonary Ewing’s sarcoma have been reported since 2010 [2–8]. In the present case, primary pulmonary Ewing’s sarcoma, which presented with clinical features of massive hemothorax, was confirmed by histopathology and immunohistochemistry.

## Case presentation

**Fig. 1:**
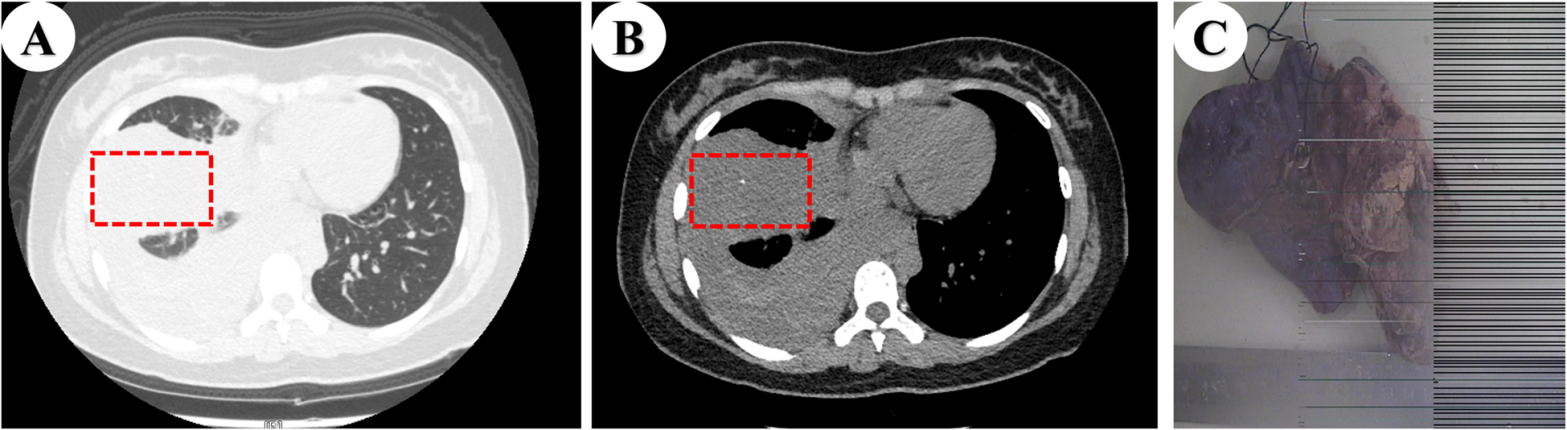
**a**, **b** Computed tomography chest axial images showing massive right pleural effusion, right lung collapse, and punctate calcification. **c** The macroscopic pathological picture of lump in the upper right lobe

A 15-year-old girl presented with gradually increasing chest pain, cough, and dyspnea for 3 days, which then worsened in 2 hours, and she was eventually treated in the emergency department. There was no significant medical history, trauma included. On physical examination, she had low-grade fever (37.6 °C), hypotension (90/58 mmHg), tachycardia (122 beats/min), pain, weakness, and pale lips. Auscultation over the chest showed reduced air entry into the right hemithorax. Hematological investigations revealed mild anemia with a hemoglobin level of 9.2 g/dl. The other biochemical investigations were within the normal range. Computed tomography (CT) was performed using a Philips Brilliance multidetector scanner for evaluation of the lung. The images demonstrated a massive right pleural effusion with the collapse of the right lung, and punctate calcification was evident in the images (Fig. 1a, b). The density of the pleural fluid was likely a result of hemothorax. The ribs and vertebrae did not show any lytic areas.

Thoracentesis was performed on the patient to determine the nature of the pleural effusion. Laboratory tests of the effusion revealed a large number of red blood cells, which appeared uncoagulated; this was suggestive of hemothorax. Progressive anemia was noted following admission, and 8-h later hematological investigations revealed marked anemia with a hemoglobin level of 8.3 g/dl. Consequently, emergency surgery was performed and acute bleeding in thoracic cavity was observed. Upon opening the right pleural cavity, approximately 2200 mL of blood was released. An anterolateral thoracotomy was performed by video-assisted thoracoscopic surgery, revealing a tumor (40 × 30 × 45 mm, Fig. 1c) in the right upper lobe with a hemorrhage on the surface. Therefore, we considered hemothorax caused by tumor rupture and hemorrhage. Another large tumor (32 × 30 × 34 mm) in the right lower lobe, and pleural tissue was invaded, but no mass tissue was seen on the pleural, suggesting that it is not the primary lesion. An analysis of frozen sections using hematoxylin and eosin staining revealed abnormal cells, indicative of malignant tumors. Two tumor tissues were completely resected, the cut surface of mass in the right upper lobe was hemorrhage and necrosis were evident. and acute bleeding in the thorax was cured.

**Fig. 2:**
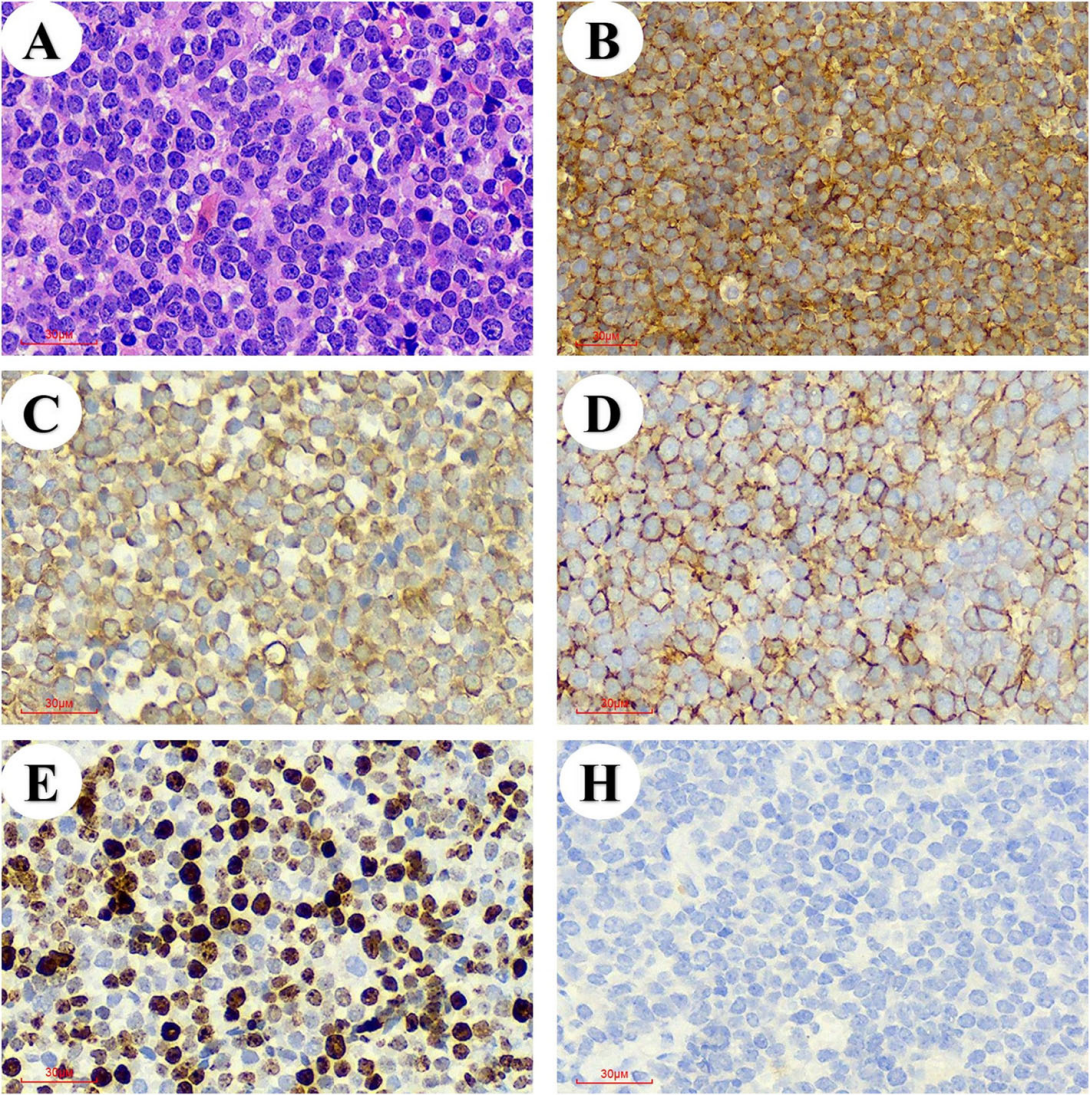
Histopathological images of **a** pleomorphic cells with narrow cytoplasm and hyperchromatic nuclei, **b** membranous immunoreactivity with CD99, **c** cytoplasmic immunoreactivity with vimentin, **d** membranous and cytoplasmic immunoreactivity with CD56, **e** > 70% positivity for Ki-67 and **f** negativity for AE1/AE3

Histopathological evaluation of the surgical biopsy specimen was conducted. Microscopy revealed nests of small pleomorphic cells arranged in sheets and clumps, with round-, oval-, or abnormally-shaped nuclei and intervening fibrous strands and engorged blood vessels. Immunohistochemical analysis showed diffuse membranous positivity for CD99, which is characteristic of Ewing’s sarcoma as well as primitive neuroectodermal tumors. Cells were positive for vimentin, CD56, and Ki-67 (more than 70%). However, cytokeratin (AE1/AE3) staining was negative (Fig. 2). The hemothorax formed due to a hemorrhage from the ruptured primary pulmonary Ewing’s sarcoma was diagnosed. A whole-body CT scan was then performed to look for possible metastasis. Subsequently, a magnetic resonance imaging brain scan was also performed. No intracranial or intra-abdominal visceral or peritoneal metastases were found, without any metastatic extra or intrathoracic skeletal involvement. Treatment with alternating courses of chemotherapy regimens of vincristine, doxorubicin, and cyclophosphamide, or ifosfamide, and etoposide were administered on postoperative day 20. After 3 cycles of chemotherapy, the patient was found to be stable at follow-up examination. No more hydrothorax or other symptoms.

## Discussion and conclusions

The present study reports a rare case of hemothorax caused by a primary pulmonary Ewing’s sarcoma. Clinical symptoms of primary pulmonary Ewing’s sarcoma depend on the site of presentation but invariably include pain and swelling of the surrounding structures due to the mass effect. Other reported symptoms and signs such as hemoptysis are site-specific. The unusual presence of spontaneous hemothorax in association with a primary pulmonary Ewing’s sarcoma, which has not been reported in the literature, prompted us to publish this case. Non-traumatic hemothorax is a rare clinical feature, which is a potentially life-threatening emergency, which necessitates urgent surgery. It generally occurs due to the erosion of a vessel caused by a pathological lesion, such as in vascular disorders, connective tissue disease, neoplasms, extramedullary hematopoiesis, endometriosis, or pulmonary sequestration [9].

Imaging characteristics of pulmonary Ewing’s sarcoma have been described in the literature, but the characterization has been limited. Typical pulmonary Ewing’s sarcoma images have a circumscribed solitary mass showing heterogeneous enhancement with non-enhancing necrotic areas within. Intralesional amorphous calcification, associated pleural effusion, and superior vein cava syndrome have also been reported [7]. The characteristics of a solitary mass cannot be found in images in critically ill patients with massive right pleural effusion and right lung collapse without enhanced CT scans. Pulmonary Ewing sarcomas tend to show large size with smooth margins and high fluorodeoxyglucose uptake [5, 10, 11]. Furthermore, fluorodeoxyglucose positron emission tomography scanning, a useful tool for initial staging, treatment response assessment, and detection of recurrence, helps exclude the possibility of pulmonary metastasis from extra-pulmonary Ewing’s sarcoma [12].

The diagnosis of extraosseous Ewing’s sarcoma is essentially histological and relies on the pathologic features of the specimen. The classical histopathological findings are that of round cells, with irregularly shaped chromatic nuclei surrounded by scanty cytoplasm; mitotic figures may be seen. The cells often show immunohistochemical positivity for various neurofilaments such as CD99, which is a sensitive diagnostic marker. Other markers include vimentin, CD56, Thyroid transcription factor-1(TTF1), cytokeratin 5/6, and caveolin 1, which facilitate differentiation from other small round cell tumors [1, 4].

In addition, electron microscopic features include a specific high nucleus-to-cytoplasm ratio with aggregated glycogen granules in the cytoplasm. Detection of *EWSR1* gene translocation or amplifcation is the most reliable marker of PNETs, including those of pulmonary origin. 85% of patients had the identification t(11;22) (q24;q12) chromosome rearrangement by fluorescent in situ hybridization and/or reverse transcription-polymerase chain reaction is used to support the diagnosis, and the remaining 15% of the patients had variants of this translocation, including 22q12, 21q12 (10% of cases) and 7p22, 17q12, 2q36 (< 1% of cases) [1, 8]. In our case, due to our laboratory conditions limitations, there is a lack of the translocation detection. The staining of CD99 was positive and the diagnosis of primary Ewing’s sarcoma was confirmed.

The treatment of Ewing’s sarcoma is aggressive, with the most effective treatment being surgical resection with combination chemotherapy and/or high-dose radiation therapy. Prognosis is mainly related to the ability to achieve disease-free surgical margins and the extent of anatomical spread to surrounding structures such as bone, pleura, and the epidural space [13]. Historically, the outcome has been poor because of the limitations of traditional chemotherapeutic approaches. The standard first-line treatment for patients with these tumors includes alternating courses of chemotherapy with the five-drug regimens of vincristine, doxorubicin, cyclophosphamide, ifosfamide, and etoposide. Pazopanib efficacy in pulmonary Ewing’s sarcoma was reported, but it is not considered a standard therapy [9]. In the present case, our patient had pleural involvement without bone extension, and was administered a combination of traditional chemotherapy (alternating courses of chemotherapy regimens of vincristine, doxorubicin, and cyclophosphamide, or ifosfamide and etoposide) post-operation. Follow-up was performed for three complete cycles of chemotherapy without any evidence of disease progression or distant metastasis.

In summary, we have described an extremely rare case of primary pulmonary Ewing’s sarcoma that presented with clinical features of massive hemothorax. Although rare, it should be considered in the differential diagnosis of large tumors of the lungs. The patient underwent surgical resection and postoperative chemotherapy. She has exhibited no sign of recurrence to date.

## Data Availability

The datasets used and/or analyzed during the current study are available from the corresponding author on reasonable request.

## Abbreviations

CT: Computed tomography
CD: Cluster of differentiation
TTF-1: Thyroid transcription factor-1
